# Investigating safe nursing care and medication safety competence in nursing students: a multicenter cross-sectional study in Iran

**DOI:** 10.1186/s12912-023-01684-0

**Published:** 2024-01-02

**Authors:** Zahra Mohebi, Mostafa Bijani, Azizallah Dehghan

**Affiliations:** 1 Student Research Committee, School of Nursing, Fasa University of Medical Sciences, Fasa, Iran; 2 Department of Medical Surgical Nursing, School of Nursing, Fasa University of Medical Sciences, 81936-13119 Fasa, Iran; 3 Noncommunicable Diseases Research Center (NCDRC), Fasa University of Medical Sciences, Fasa, Iran

**Keywords:** Clinical competence, Patient safety, Nursing care, Nursing, Students, Medication

## Abstract

**Background:**

Medication safety competence is very important as one of the clinical skills among nursing students to provide safe nursing care. The lack of medication safety competence in nursing students leads to occurrence of medication errors subsequently jeopardizing patient safety. Thus, the present study was conducted to investigate safe nursing care and medication safety competence among nursing students in the south of Iran.

**Methods:**

A descriptive cross-sectional multicenter study was conducted from September to December 2022. The research population included nursing students of three universities of medical sciences in Fars Province, Southern Iran. A total of 310 nursing students who were selected through convenience sampling participated in the study. The data collection instruments consisted of a demographics survey, Medication Safety Competence Scale (MSCS), and a Safe Nursing Care Scale (SNCS). The collected data were analyzed using descriptive statistics (absolute and relative frequency, mean and standard deviation) and inferential statistics (Independent t-test, Analysis of variance and Pearson correlation coefficient). The data were analyzed in SPSS 23 and the level of significance was considered 0.05.

**Results:**

The mean age of the participants was 22.53 ± 1.69 years. The total mean scores for medication safety competence and safe nursing care were found to be 111.97 ± 11.85 and 105.12 ± 11.64, respectively. There was a statistically significant positive correlation between safe nursing care and medication safety competence (r = 0.084, *P* < 0.001).

**Conclusion:**

The mean scores of nursing students’ medication safety competence and safe nursing care were at an average level. To maintain patient safety, nursing instructors and managers are recommended to employ appropriate strategies to improve medication safety competence and safe nursing care in nursing students.

## Introduction

The high risk of physical injuries and financial burden on patients have turned patient safety into a global concern [1]. According to the National Institutes of Health, safety in healthcare environments includes preventing errors and the side effects of treatments as well as not harming patients in the course of medical procedures [1–2]. Preservation of patient safety was underscored inf a report by the American National Institutes of Health in 1999. Entitled “To Err is Human,” the report addressed the rate of medical errors in the U.S. and drew the attention of researchers and healthcare experts to the subject of patient safety more than ever [2]. One of the primary objectives of a medical center is to provide safe and effective care to patients, where nurses, as the frontline workers in caregiving, are a major group in fulfilling this goal [3]. Healthcare services are provided in environments which are influenced by a variety of factors, including patients, caregivers, patients’ families, equipment, and managers’ policies. When these factors interfere, the consequences are dangerous and unpredictable [4]. Every year, millions of patients in the world suffer disabling injuries or death due to unsafe healthcare, and at least one out of ten patients is injured despite receiving care in a high-tech hospital [5]. Nevertheless, approximately 50% of the national running costs are claimed by healthcare services in hospitals [6].

In addition to the above-mentioned physical harms, the financial burden of caregivers’ failure to comply with the principles of patient safety is another issue in healthcare systems. World Health Organization (WHO) reports that unsafe care augments medical costs by increasing the length of stay, lowering incomes, causing disabilities, and imposing legal expenses; in some countries, these expenses approach billions of dollars [5].

So far, many studies have addressed different aspects of safe nursing care and the associated factors, e.g. medical errors, inaccurate reporting, and the culture of safety. Another important factor in safe nursing care is medication safety. Adherence to medication instructions is an essential part of treatment as well as patient safety and care [7]. The goal of safe medication therapy is to provide comprehensive medical services to patients and ensure the logic of prescribed medication plus improvement in the patients’ health status [8]. Medication safety also means protecting patients from accidental injuries, avoiding any preventable complications and side effects, along with fulfilling maximum medication effect [9]. Medication safety involves accurate evaluation of patients, selection, and administration of medication in the right dose and at the right time, attention to cases contraindications, side effects, and drug interactions [10].

Medication errors are among the most common type of clinical errors [11]. Approximately, one third of adverse drug events are due to medication errors [12], with negative consequences for patients, nurses, and other members of the healthcare team, and decline in the quality of care [13].

Despite system-based technological advances, nearly 100 thousand patients are harmed because of preventable medication administration errors in healthcare centers every year [14]. According to a report on medication safety released by Federal University of São Paulo, medication errors or the side effects of medications account for 7% of hospitalizations in the healthcare system, and every year 44 to 98 thousand deaths are caused by medication errors in the U.S., which costs the healthcare system 17 to 29 billion dollars [15]. Medication errors can endanger the patients’ safety, which is the main concern of healthcare systems worldwide and could lead to mortality, morbidity, complications, and prolonged length of stay in health care [16].

The results of the study by Dehvan et al. (2021), which was conducted as a systematic review and meta-analysis, revealed that the prevalence of medication errors in nursing students in different countries varies within 10–80%. According to the results of the same study, the prevalence of medication errors in nursing students in Iran has been reported as 39.68% [17]. Perhaps, one of the reasons for these medication errors is the lack of medication safety competence in nursing students. Nursing students in the clinical education environment are exposed to a range of medical errors, especially medication errors [18]. Medication safety competence is very important as one of the clinical competencies in nursing students to provide safe nursing care. The lack of medication safety competence in nursing students leads to the occurrence of medication errors thereby jeopardizing patient safety; in some cases, it can lead to the death of the patients [19]. The goal of assessing safe nursing care and medication safety competence is to pave the way for improving them, provide safer care, and protect the society from preventable harms and unwanted complications [20]. Evidence shows that nursing students are still making medication errors while working in clinical settings. Medication dosage calculation errors and administration errors continue to occur even with new guidelines, technology, polices, and education [21–22].

For investigating the medicine safety competence in nursing students as well as identifying the strengths and weaknesses of the nursing students’ performance in medicine safety and in turn safe nursing care, it is necessary to use a standard instrument which specifically evaluates Medication Safety Competence Scale (MSCS). Given the importance of the subject, and that few studies have been conducted in this area, the present study is also suggested to be carried out in different countries to develop the nursing knowledge. Also, nursing instructors and managers can utilize the results of this study to improve the medication safety competence and safe care in nursing students. Accordingly, the present study was conducted to investigate safe nursing care and medication safety competence in nursing students.

## Methods

### Study design, setting, and participants

This descriptive cross-sectional multicenter study was conducted from September to December 2022 based on the STROBE Statement (Strengthening the Reporting of Observational Studies in Epidemiology) [23]. (Supplementary file 1: STROBE checklist). The research population included nursing students with bachelor’s degree in three universities of medical sciences in Fars Province, Southern Iran (n = 310).

The inclusion criteria were a bachelor’s degree nursing students, having passed a course on theoretical and practical pharmacology, and willingness to participate in the study. The exclusion criteria included the unwillingness to continue participating in the study for any reason. For sampling, the first author (ZM) attended the school of nursing affiliated with a university of medical sciences in the Fars Province, Southern Iran, and asked the nursing students who met the inclusion criteria to fill out the questionnaires.

The participants were selected using convenience sampling. The sample size was determined using the results of a study by Khalili et al. [7]. and the following formula. In this formula, Z represents confidence level, which was set at 95% in this study, while d represents acceptable degree of difference, which was set at 5%. In the afore-mentioned study, the extent of drug interaction errors was reported to be 9%; thus, the minimum sample size was calculated to be 126. To boost the credibility of the findings, the researchers enrolled 310 subjects.

1 $$ $$n = \frac{{{Z^2}.P(1 - P)}}{{{d^2}}}$$ $$

### Evaluation tool and data collection

#### Demographic features

The demographic survey captured the participants’ age, gender, marital status, and academic semester.

#### Medication safety competence scale (MSCS)

Developed and validated by Seomun and Park in South Korea, the MSCS consists of 36 items which are scored on a 5-point Likert scale (ranging from never to always). The score range of the scale is 36–180, with scores of 36–90 indicating poor medication safety competence, 91–150 average medication safety competence, and 151–180 satisfactory medication safety competence. This scale consists of six factors, namely medication management and patient assessment, improvement of safety problems in the medication process, management of effecting factors, management of safety risks, multidisciplinary collaboration, and responsibility as a professional nurse. The Cronbach’s alpha of the scale was reported to be 0.96 as well as between 0.77 and 0.91 for its different factors [24].

The MSCS was translated and validated by Mohammadi et al. in Iran (2023). Exploratory factor analysis (EFA) results revealed that the factor loading of the 36 items ranged from 0.72 to 0.87 and that all of them were significant. Confirmatory factor analysis (CFA) results indicated goodness of fit of the data (χ2/df = 7, RMSEA = 0.01, CFI = 0.96, NFI = 0.95, and TLI = 0.97). To test the reliability of the scale, the researchers measured its internal homogeneity and found the Cronbach’s alpha of the whole instrument to be 0.96. Thus, the Persian version of MSCS for nurses is adequately valid and reliable [25].

#### Safe nursing care scale (SNCS)

The SNCS was developed by Rashvand et al. The reliability of the scale was validated with a Cronbach’s alpha of 0.97. This instrument consists of 32 items which fall into four parts: part 1 (16 items) assesses nursing skills, part 2 (4 items) addresses evaluation of the psychological safety of the patient, part 3 (7 items) deals with evaluation of the physical safety of the patient, and part 4 (5 items) evaluates the nurse respondent’s teamwork performance. All items are scored on a 5-point Likert scale: never = 1, rarely = 2, sometimes = 3, usually = 4, and always = 5. This scale also determines the loading of each item: items 14, 18, 19, 20, and 32 have a loading of 1; items 2, 3, 4, 5, 7, 10, 11, 12, 13, 15, 16, 17, 21, 26, and 30 have a loading of 2; items 1, 6, 8, 9, 23, 24, 25, 27, 29, and 31 have a loading of 3; and items 22 and 28 have a loading of 4. Thus, the score for each item was multiplied by its loading, and the result was used for analysis. A score of 73 to 170 indicates poor, 171 to 267 average, and 268 to 365 satisfactory performance [26].

### Statistical analysis

The collected data were analyzed using descriptive statistics (absolute and relative frequency, mean and standard deviation) plus inferential statistics (Independent t-test, Analysis of variance and Pearson correlation coefficient). The data were analyzed using SPSS 23, and the level of significance was set at 0.05.

### Ethical considerations

All participants gave written informed consent to participate in the study. The present study was conducted based on the principles of the revised Declaration of Helsinki, which is a statement of ethical principles that directs physicians and other participants in medical research involving human subjects. The participants were assured of their anonymity and confidentiality of their information. The study was also approved by the Institutional Research Ethics Committee of Fasa University of Medical Sciences, Fasa, Iran (Ethical code: (IR.FUMS.REC.1401.109)

## Results

Among the 310 bachelor’s degree nursing students who participated in the study, the youngest was 20, while the oldest was 30 years old. The mean age of the participants was 22.53 ± 1.69. Table 1 reports the other demographic characteristics of the students. The participants’ total mean score of medication safety competence was 111.97 ± 11.85; the lowest mean score (9.54 ± 1.85) belonged to the domain of responsibility as a professional nurse and the highest mean score (27.89 ± 3.50) to the management of influential factors (Table 2). The participants’ total mean score of safe nursing care was 105.12 ± 11.64; the lowest mean score (13.08 ± 2.38) was related to the domain of psychological safety and the highest mean score (50.74 ± 6.97) to the domain of nursing skills (Table 3).

**Table 1:** Frequency distribution of the nursing students’ demographics

Variable		Number	Percentage
Gender	Male	89	28.70
	Female	221	71.30
Academic Semester	4	63	20.32
	5	88	28.37
	6	90	29.03
	7	29	9.35
	8	40	12.90
Marital status	Single	261	84.20
	Married	33	10.64
	Other	16	5.16

**Table 2:** Mean and standard deviation of the nursing students’ medication safety competence scores

Scale and dimension	mean	SD
Medication management and patient assessment	24.86	5.60
Elimination of safety problems in the medication process	18.05	3.73
Management of effecting factors	27.89	3.50
Management of safety risks	19.06	3.44
Multidisciplinary collaboration	12.54	2.23
Responsibility as a professional nurse	9.54	1.85
**Total medication safety score**	**111.97**	**11.85**

**Table 3:** Mean and standard deviation of the nursing students’ safe nursing care scores

Scale and dimension	mean	SD
Nursing skills	50.74	6.97
Psychological safety	13.08	2.38
Physical safety	23.56	4.72
Teamwork	17.73	3.30
**Total safe nursing care score**	**105.12**	**11.64**

As outlined by Tables 4 and 95.80% and 96.12% of the surveyed students had average medication safety competence and safe nursing care scores, respectively. The results indicated that none of the students’ score of safe nursing care was satisfactory, and only 13 (4.20%) students’ scores in medication safety competence were at a satisfactory level. The results also revealed a statistically significant direct correlation between safe nursing care and medication safety competence as well as across all their dimensions (*P* < 0.05) (Table 5).

**Table 4:** Frequency distribution of the nursing students’ medication safety competence and safe nursing care

Variable		Number	Percentage
Medication safety competence	Poor	0	0
	Average	297	95.80
	Satisfactory	13	4.20
	Total	310	100
Safe nursing care	Poor	12	3.88
	Average	298	96.12
	Satisfactory	0	0
	Total	310	100

**Table 5:** The correlation between the nursing students’ scores for medication safety competence and safe nursing care according to their dimensions

Medication safety competence and its dimensions	Safe nursing care and its dimensions				
	nursing skills	psychological safety	physical safety	teamwork	Total safe nursing care score
medication management and patient assessment	r = 0.249 *P* < 0.001	r = 0.008 *P* = 0.898	r = 0.356 *P* < 0.001	r = 0.395 *P* < 0.001	r = 0.168 *P* = 0.008
improvement of safety problems in the medication process	r = 0.143 *P* = 0.029	r = 0.124 *P* = 0.051	r = 0.296 *P* < 0.001	r = 0.263 *P* < 0.001	r = 0.195 *P* = 0.002
management of effecting factors	r = 0.004 *P* = 0.095	r = 0.135 *P* = 0.033	r = 0.048 *P* = 0.051	r = 0.015 *P* = 0.0815	r = 0.043 *P* = 0.0498
management of safety risks	r = 0.201 *P* < 0.001	r = 0.112 *P* = 0.076	r = 0.061 *P* = 0.034	r = 0.163 *P* < 0.001	r = 0.204 *P* < 0.001
multidisciplinary collaboration	r = 0.123 *P* = 0.053	r = 0.150 *P* = 0.018	r = 0.197 *P* = 0.002	r = 0.185 *P* = 0.003	r = 0.228 *P* < 0.001
responsibility as a professional nurse	r = 0.207 *P* < 0.001	r = 0.164 *P* = 0.009	r = 0.028 *P* = 0.660	r = 0.138 *P* = 0.029	r = 0.203 *P* < 0.001
Total medication safety score	r = 0.028 *P* < 0.001	r = 0.077 *P* = 0.022	r= -0.286 *P* < 0.001	r=-0.210 *P* = 0.001	r = 0.084 *P* < 0.001

r: Pearson’s correlation coefficient

## Discussion

The present study was conducted to explore safe nursing care and medication safety competence in nursing students in 2022 to reduce medication errors, and in turn, improve the quality as well as safety of the care provided to patients.

In this study, the findings revealed that there was a significant relationship between safe nursing care and medication safety competence in nursing students. In other words, the ability of nursing students to administer drugs safely determines the quality of services and supports the patient safety. In our study, most nursing students were at an average level in terms of safe nursing care and medication safety competence, which indicates a relatively high level of knowledge and skill in nursing students; more learning experiences are required during the academic courses, since providing safe nursing care and maintaining the safety of patients are very important points students should learn from the very beginning.

The findings of the present study indicated that multidisciplinary cooperation and teamwork, as important dimensions of medication safety competence, directly correlate with safe nursing care. Similarly, a study by Franklin et al. showed that providing nursing care as a team and collective understanding would improve the patient’s safety and bring about other valuable results [27]. Also, according to a study by Ginsberg et al., caregivers’ skills for management of group activities facilitate safer and more effective care of patients, promote patient-centered care, and encourage the personnel to have a more positive attitude [28]. The findings of the present study also demonstrated that there was a significant direct correlation between nursing skills on the one hand and medication management and evaluation of patients on the other. The results of a study by Smeulers et al. are in the same line with these findings; further, safe provision and administration of medication, clinical reasoning and nurses’ experiences are essential to medication safety. In addition, awareness of their work hazards and conditions would impact the nurses’ ability to work safely [29]. Also, according to a study by Rohde, nurses play a key role in safe medication administration; nurses should use their knowledge, experience, and skills to support medication safety according to patients’ conditions and organizational procedures [30].

In the current study, it was also found that there was a statistically significant direct correlation between elimination of safety problems in the medication process and patients’ physical plus psychological safety. In other words, elimination of safety problems in the medication process contributes to patient physical and psychological safety. Maintaining patient safety is one of the primary professional and ethical responsibilities of all care providers. Obviously, although nothing is more discordant to healthcare principles like harming a patient, medical interventions and procedures are not always completely safe and occurrence of medical errors and other events which put patient safety at risk is a real possibility. Critical conditions would promote the incidence of medication errors, which in turn undermine the patient physical and psychological safety [31]. A study by Kazemi reported that a better patient safety culture correlated with fewer medication errors and better preservation of patient safety. Thus, healthcare and hospital authorities should set up training programs and workshops to improve the patient safety culture [32].

The present study also found that having a sense of responsibility in nurses improved all aspects of safe nursing care. Nurses form the foundation of improvement in healthcare services; therefore, their performance determines achievement of organizational objectives; nurses’ sense of responsibility plays a crucial role in accomplishment of the goals of the healthcare system and nursing. According to a study by Ghorbani et al., there is a significant direct correlation between the nurses’ sense of responsibility and the quality of care provided in hospitals; thus, educating the personnel in responsibility-related areas, responsiveness, and communication skills is beneficial and is an effective way to improving the quality of services [33]. Similarly, in their study, Mohammadi et al. found a direct correlation between the quality of education and sense of responsibility in nurses. Increasing the quality of education in nursing schools and raising responsible nursing students can enhance the students’ efficiency in clinical environments and promote safe nursing care [34].

The findings of this study also revealed that management of the influential factors in medication safety had a positive impact on all the dimensions of safe nursing care, i.e. nursing skills, psychological safety of the patient, physical safety of the patient, and teamwork. Likewise, the results of a study carried out by Huang and Chen demonstrated that nursing safety management can significantly enhance the quality of nursing and prevent unwanted hazards [35]. As to safe nursing care, most nursing students were found to be at an average level, and none of them obtained a satisfactory score. Providing safe care lies at the core of high-quality nursing care. In the present study, the nursing students’ highest safe care score belonged to nursing skills, followed by physical safety of the patient, teamwork, and finally psychological safety of the patient. However, in studies by Kalantari [36] and Fotoohi [37], the best aspect of the nurses’ performance in terms of safety was related to physical safety of the patient, followed by psychological safety, teamwork, and finally nursing skills, which is not consistent with the findings of the present study.

As with safe nursing care, medication safety competence was found to be at an average level in many of the nursing students and only a few (5.2%) obtained satisfactory scores. Pharmacotherapy, which is known as the most common form of treatment, is a domain where most medical errors occur. Thus, medication safety competence should be stressed in nursing requirements. The results of a study by Fernandeze et al. are in line with these findings and verify that nursing students are prone to medication errors in field training programs, which poses a threat to patients’ safety. Therefore, it is essential that measures should be taken to manage and reduce such errors [38].

### Limitations

In the present study, only undergraduate nursing students participated; it is recommended that the study be conducted on nursing graduate students as well. Also, this study was conducted in the south of Iran; it is also suggested that research be carried out in other regions of Iran and other countries as well. Also, given that few studies have been conducted in the field of medication safety competence and safe nursing care in nursing students, the authors had limitations in comparing the results of this study with those of other investigations.

### Strengths

This is the first study in Iran conducted to investigate safe nursing care and medication safety competence in nursing students, which is a novelty. Also, one of the strengths of this study has been the use of a comprehensive and specific questionnaire (Medication Safety Competence Scale) for assessing the competence of pharmaceutical safety, which has been translated and validated in Iran.

## Conclusion

The findings of the present study revealed a significant direct correlation between medication safety competence and safe nursing care, indicating that improvement in different domains of medication safety competence can contribute to safe nursing care. In the current study, the nursing students’ medication safety competence and safe nursing care mean scores were at an average level. To maintain the patient safety, nursing instructors and managers are recommended to employ appropriate strategies to improve medication safety competence and safe nursing care in nursing students. It appears that employment of better learning and teaching approaches to medication prescription and administration can improve the nursing students’ professional performance as well as contribute to the safety and quality of care.

## Data Availability

The datasets generated and/or analysed during the current study are not publicly available due to the necessity to ensure participant confidentiality policies and laws of the country but are available from the corresponding author on reasonable request.

## Abbreviations

MSCS: Medication Safety Competence Scale
SNCS: Safe Nursing Care Scale
